# Solvable Model for the Linear Separability of Structured Data

**DOI:** 10.3390/e23030305

**Published:** 2021-03-04

**Authors:** Marco Gherardi

**Affiliations:** 1 Department of Physics, Università degli Studi di Milano, via Celoria 16, 20133 Milano, Italy; marco.gherardi@mi.infn.it; 2 Istituto Nazionale di Fisica Nucleare Sezione di Milano, via Celoria 16, 20133 Milano, Italy

**Keywords:** linear separability, storage capacity, data structure

## Abstract

Linear separability, a core concept in supervised machine learning, refers to whether the labels of a data set can be captured by the simplest possible machine: a linear classifier. In order to quantify linear separability beyond this single bit of information, one needs models of data structure parameterized by interpretable quantities, and tractable analytically. Here, I address one class of models with these properties, and show how a combinatorial method allows for the computation, in a mean field approximation, of two useful descriptors of linear separability, one of which is closely related to the popular concept of storage capacity. I motivate the need for multiple metrics by quantifying linear separability in a simple synthetic data set with controlled correlations between the points and their labels, as well as in the benchmark data set MNIST, where the capacity alone paints an incomplete picture. The analytical results indicate a high degree of “universality”, or robustness with respect to the microscopic parameters controlling data structure.

## 1. Introduction

Linear classifiers are quintessential models of supervised machine learning. Despite their simplicity, or possibly because of it, they are ubiquitous: they are building blocks of more complex architectures, for instance, in deep learning and support vector machines, and they provide testing grounds of new tools and ideas in learning theory and statistical mechanics, in both the study of artificial neural networks and in neuroscience [1,2,3,4,5,6,7,8,9]. Recently, interest in linear classifiers was rekindled by two outstanding results. First, deep neural networks with wide layers can be well approximated by linear models acting on a well defined feature space, given by what is called “neural tangent kernel” [10,11]. Second, it was discovered that deep linear networks, albeit identical to linear classifiers for what concerns the class of realizable functions, allow it to reproduce and explain complex features of nonlinear learning and gradient flow [12].

In spite of the central role that linear separability plays in our understanding of machine learning, fundamental questions still remain open, notably regarding the predictors of separability in real data sets [13]. How does data complexity affect the performance of linear classifiers? Data sets in supervised machine learning are usually not linearly separable: the relations between the data points and their labels cannot be expressed as linear constraints. The first layers in deep learning architectures learn to perform transformations that enhance the linear separability of the data, thus providing downstream fully-connected layers with data points that are more adapted for linear readout [14,15]. The role of “data structure” in machine learning is a hot topic, involving computer scientists and statistical physicists, and impacting both applications and fundamental research in the field [16,17,18,19,20,21,22].

Before attempting to assess the effects of data specificities on models and algorithms of machine learning, and, in particular, on the simple case of linear classification, one should have available (i) a quantitative notion of linear separability and (ii) interpretable parameterized models of data structure. Recent advances, especially within statistical mechanics, mainly focused on point (ii). Different models of structured data have been introduced to express different properties that are deemed to be relevant. For example, the organization of data as the superposition of elementary features (a well-studied trait of empirical data across different disciplines [23,24,25]) leads to the emergence of a hierarchy in the architecture of Hopfield models [26]. Another example is the “hidden manifold model”, whereby a latent low-dimensional representation of the data is used to generate both the data points and their labels in a way that introduces nontrivial dependence between them [19]. An important class of models assumes that data points are samples of probability distributions that are supported on extended object manifold, which represent all possible variations of an input that should have no effect on its classification (e.g., differences in brightness of a photo, differences in aspect ratio of a handwritten digit) [27]. Recently, a useful parameterization of object manifolds was introduced that is amenable to analytical computations [28]; it will be described in detail below. In a data science perspective, these approaches are motivated by the empirical observation that data sets usually lie on low-dimensional manifolds, whose “intrinsic dimension” is a measure of the number of latent degrees of freedom [29,30,31].

The main aims of this article are two: (i) the discussion of a quantitative measure of linear separability that could be applied to empirical data and generative models alike; and, (ii) the definition of useful models expressing nontrivial data structure, and the analytical computation, within these models, of compact metrics of linear separability. Most works concerned with data structure and object manifolds (in particular, Refs. [8,27,28]) focus on a single descriptor of linear separability, namely the storage capacity $\alpha_{c}$. Informally, the storage capacity measures the maximum number of points that a classifier can reliably classify; in statistical mechanics, it signals the transition, in the thermodynamic limit, between the SAT and UNSAT phases of the random satisfiability problem related to the linear separability of random data [32]. Here, I will present a more complete description of separability than the sole storage capacity (a further motivation is the discovery, within the same model of data structure, of other phenomena lying “beyond the storage capacity” [33]).

## 2. Linear Classification of Data

Let us first review the standard definition of linear separability for a given data set. In supervised learning, data are given in the form of pairs $\left(\xi_{\mu },\sigma_{\mu }\right)$, where $\xi_{\mu }\in R^{n}$ is a data point and $\sigma_{\mu }=\pm 1$ is a binary label. We focus on dichotomies, i.e., classifications of the data into two subsets (hence, the binary labels); of course, this choice does not exclude datasets with multiple classes of objects, as one can always consider the classification of one particular class versus all the other classes. Given a set of points $X={\left\{\xi_{\mu }\right\}}_{\mu =1,\ldots ,m}$, a dichotomy is a function $\phi :X\to {\left\{-1,+1\right\}}^{m}$. A data set ${\left\{\left(\xi_{\mu },\sigma_{\mu }\right)\right\}}_{\mu =1,\ldots ,m}$ is linearly separable (or equivalently the dichotomy $\phi \left(\xi_{\mu }\right)=\sigma_{\mu }$, μ=1,…,m, is linearly realizable) if there exists a vector $w\in R^{n}$, such that

(1) $$\mathrm{sgn}\left(\sum_{i=1}^{n}w_{i}\cdot {\left(\xi_{\mu }\right)}_{i}\right)=\sigma_{\mu },\;\mu =1,\ldots ,m,$$

where ${\left(\xi_{\mu }\right)}_{i}$ is the *i*th component of the μth element of the set. In the following, I will simply write $w\cdot \xi_{\mu }$ for the scalar product appearing in the sgn function when it is obvious that *w* and $\xi_{\mu }$ are vectors.

In machine learning, the left hand side of Equation (1) is the definition of a linear classifier, or perceptron. The points *x*, such that w·x=0 define a hyperplane, which is the separating surface, i.e., the boundary between points that are assigned different labels by the perceptron. By viewing the perceptron as a neural network, the vector *w* is the collection of the synaptic weights. “Learning” in this context refers to the process of adjusting the weight vector *w* so as to satisfy the *m* constraints in Equation (1). Because of the fact that the sgn function is invariant under multiplication of its argument by a positive constant, I will always consider normalized vectors, i.e., both the weight vector *w* and data points ξ will lie on the unit sphere.

A major motivation behind the introduction of the concept of data structure and the combinatorial theory that is related to it (reviewed in Section 5 and Section 6 below) is the fact that the definition of linear separability above is not very powerful per se. Empirically relevant data sets are usually not linearly separable. Knowing whether a data set is linearly separable does not convey much information on its structure: crucially, it does not allow quantifying “how close” to being separable or nonseparable the data set really is. To fix the ideas, let us consider a concrete case: the data set MNIST [34]. MNIST is a collection of handwritten digits, digitized as 28×28 greyscale images, each labelled by the corresponding digit (“0” to “9”). I will use the “training” subset of MNIST, containing 6000 images per digit. To simplify the discussion, I will mainly focus on a single dichotomy within MNIST: that expressed by the labels “3” and “7”. The particular choice of digits is unimportant for this discussion; I will give an example of another dichotomy below, when subtle differences between the digits can be observed.

One may ask the question as to whether the MNIST training set, as a whole, is linearly separable. However, the answer is not particularly informative: the MNIST training set is not linearly separable [34]. But how unexpected is this answer? Can we measure the surprise of finding out a given training set is or is not linearly separable? Intuitively, there are three different properties of a data set that facilitate or hinder its linear separability: size, dimensionality, and structure.

- **Size.** The number of elements *m* of a data set is a simple indication of its complexity. While a few data points are likely linearly separable, they convey little information on the “ground truth”, the underlying process that generated the data set. On the contrary, larger data sets are more difficult to classify, but the information that is stored in the weights after learning is expected to be more faithful to the ground truth (this is related to the concept of “sample complexity” in machine learning [35]).
- **Dimensionality.** There are two complementary aspects when considering dimensionality in a data oriented framework. First, the embedding dimension is the number of variables that a single data point comprises. For instance, MNIST points are embedded in $R^{784}$, i.e., each of them is represented by 784 real numbers. The embedding dimension is *n* in Equation (1); therefore, *n* is also the number of degrees of freedom that a linear classifier can adjust to find a separating hyperplane. Hence, one expects that a large embedding dimension promotes linear separability. Second, the data set itself does not usually uniformly occupy the embedding space. Rather, points lie on a lower-dimensional manifold, whose dimension *d* is called the intrinsic dimension of the data set. The concept of general position discussed below is related to the intrinsic dimension; however, beyond that, I will not explicitly consider this type of data complexity in this article (for analytical results on the linear separability of manifolds of varying intrinsic dimension, see [27]).
- **Structure.** As I will show in a moment, the effects of size and dimensionality on linear separability are easily quantified in a simple null model. Data structure, on the other hand, has proved more challenging, and it is the main focus of the theory described here. There is no single definition of data structure; different definitions are useful in different contexts. A common characterization can be given like this: data have structure whenever the data points $\xi_{\mu }$ and their labels $\sigma_{\mu }$ are not independent variables. I will specify a more precise definition in Section 5. Intuitively, the data structure can both promote or preclude linear separability. If points that are close to one another tend to have the same label then linear separability is improved; if, instead, there are many differently labeled points in a small region of space, then linear separability is obstructed.

Let us get back to the question “how surprising is it that MNIST is not linearly separable?”. This question should be answered by at least taking into account the first two properties described above, the size of the data set and its dimensionality, which are readily computed from the raw data. In fact, the surprise, i.e., the divergence from what is expected based on size and dimensionality, may be interpreted as a beacon of the third property: data structure. I will show in the next section that the answer to our question is “exceedingly unsurprising”. Yet, a slightly modified question will reveal that MNIST, albeit unremarkable in it not being linearly separable, is exceptionally structured.

## 3. Null Model of Linear Separability

Let us consider a null model of data that fixes the dimension *n* and the size *p*. I use a different letter (*p* instead of *m*), because it will be useful below to have two different symbols for the size of the whole data set (*m*) and for the size of its subsets. Consider a data set $Z_{p}={\left\{\left(\xi_{\mu },\sigma_{\mu }\right)\right\}}_{\mu =1,\ldots ,p}$, where the vectors $\xi_{\mu }$ are random independent variables that are uniformly distributed on the unit sphere, and the labels $\sigma_{\mu }$ are independent Bernoulli random variables (also independent from every $\xi_{\mu }$). These choices are suggested by a maximum entropy principle, when only the parameters *m* and *n* are fixed. What is the probability that a data set generated by this model is linearly separable? This problem was addressed and solved more than half a century ago [36,37,38]; In Section 6 I will describe an analytical technique that allows this computation. The fraction of dichotomies of a random data set that are linearly realizable is

(2) cn,p=21−p∑i=0n−1p−1i,

where ·· is the binomial coefficient. Thus, a random (uniform) dichotomy has probability $c_{n,p}$ of being linearly realizable. In this article, I will refer to the probability $c_{n,p}$ as the separability, or probability of separation. A related quantity is the number of dichotomies $C_{n,p}=2^{p}c_{n,p}$ (here, $2^{p}$ is the total number of dichotomies of *p* points).

**Figure 1:**
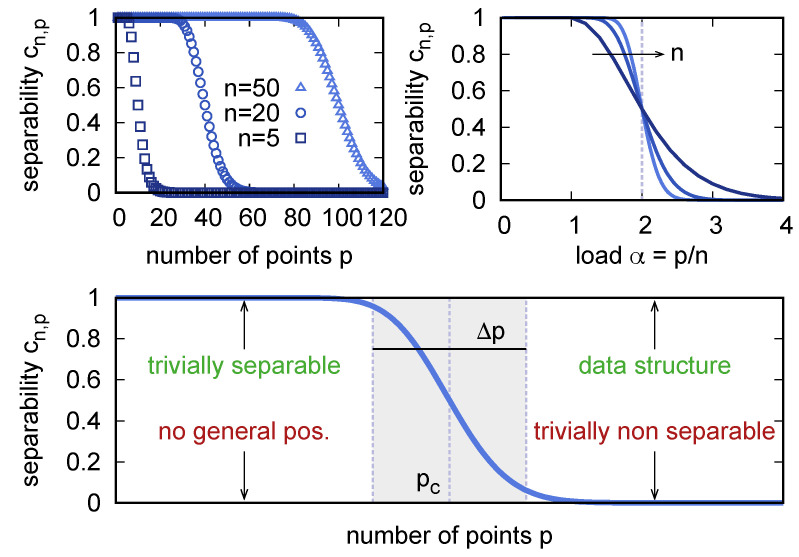
(**Top left**) The probability of separation, Equation (2), as a function of the number of points *p* for three values of the embedding dimension *n*. (**Top right**) As a function of the load α=p/n, the probability of separation converges, for large *n*, to a step function. (**Bottom**) Depending on the values of *n* and *p*, a data set being separable or nonseparable can convey information about its structure. The location $p_{c}$ and the width Δp of the transition region are the two main descriptors of the shape of a separability curve.

Figure 1 shows the sigmoidal shape of $c_{n,p}$ as a function of *p* at fixed *n*. The separability is exactly equal to 1 up to p=n (which pinpoints what is known as the Vapnik–Chervonenkis dimension in statistical learning theory [35]), and it stays close to 1 up to a critical value $p_{c}$, which increases with *n*. At $p_{c}$, the curve steeply drops to asymptotically vanishing values, the more abruptly the larger is *n*. Rescaling the number of points *p* with the dimension *n* yields the load α=p/n. As a function of α, the probability of separation has the remarkable property of being equal to 1/2 at the critical value (that is known as the storage capacity) $\alpha_{c}=p_{c}/n=2$, independently of *n*. Such an absence of finite size corrections to the location of the critical point is an unusual feature, which will be lost when we consider structured data below. In the large-*n* limit, $c_{n,\alpha n}$ converges to a step function that transitions from 1 to 0 at $\alpha_{c}$.

**Figure 2:**
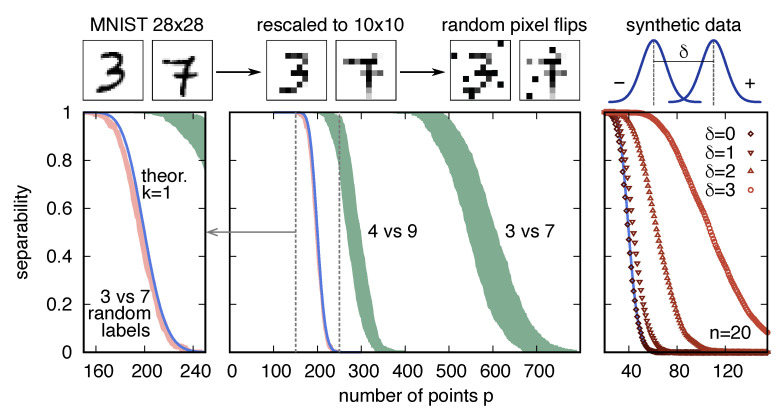
Linear separability (*y* axis) for subsets of varying size *p* (*x* axis), computed in a modified MNIST data set, generated by downscaling and applying multiplicative noise (**left** and **center** panels), and in synthetic data sets generated from a mixture of two normal distributions (**right** panel). (**Left** panel) If the labels are reshuffled, MNIST data (pink area) almost perfectly follow the prediction of the null model (blue line). (**Center** panel) The separabilities of two representative dichotomies in the data set (digits “4” versus “9”, and digits “3” versus “7”) are far removed from the null model, as is apparent from the location (and the width) of their transition regions (green areas). The shaded areas denote the 95% variability intervals. (**Right** panel) By increasing the distance δ between the means of the two Gaussian distributions that define the synthetic data set (here in n=20 dimensions), the separability increases. For δ=0 (squares), one recovers the prediction of the null model (blue line). Error bars (not shown) are approximately the same size as the symbols.

How large is the probability of separation $c_{n,m}$ that is given by Equation (2) when one substitutes the sample size *m* = 12,000 and the dimensionality n=784, i.e., those of the dichotomy “3”/“7” in the data set MNIST? The probability, as anticipated, is utterly small, less than $10^{-2000}$: it should be no surprise that MNIST is not linearly separable. This comparison is not completely fair, because of the assumption, underlying Equation (2), of general position. The concept of general position is an extension of that of linear independence, which is useful for sets larger than the dimension of the vector space. A set *X* of vectors in $R^{n}$ is in a general position if there is no linearly dependent subset $X^{\prime }\subseteq X$ of cardinality less than or equal to *n*. MNIST is quite possibly not in general position. To make sure that it is, I downscaled each image to 10×10 pixels and only considered 1000 images per class (to allow for faster numerical computations), and applied mild multiplicative random noise, by flipping 5% of the pixels around the middle grey value (see Figure 2); I will refer to this modified dataset as “rescaled MNIST”. Running the standard perceptron algorithm on rescaled MNIST did not show signs of convergence after $10^{5}$ iterations, which indicated that the data set is likely not linearly separable. For m=2000 and n=100, the separability $c_{n,m}$ is less than $10^{-400}$.

The null model provides a simple concise interpretation of the linear separability of a given data set, given its size *m* and dimensionality *n*, in terms of 5 possible outcomes (see Figure 1, bottom panel):

1. The set is linearly separable and it lies in the region where $c_{n,m}\approx 1$. Separability here is trivial: almost all data sets are separable in this region, provided that the points are in general position.
2. The set is not linearly separable and it lies in the region where $c_{n,m}\approx 1$. The only way this can happen for m≤n is if the points are not in a general position. For m>n, but still in this region, the lack of separability could also be attributed to a non-trivial data structure.
3. The set is not linearly separable and it lies in the region where $c_{n,m}\approx 0$. Almost no dichotomy is linearly realizable in this region; therefore, the lack of separability is trivial here.
4. The set is linearly separable and it lies in the region where $c_{n,m}\approx 0$. This situation is the hallmark of data structure. The fact that the data set happens to represent one of the few dichotomies that are linearly realizable in this region indicates a non-null dependence between the labels and the points in the data set.
5. The set lies in the region where $c_{n,m}$ is significantly different from 0 and 1. Here, knowing that a data set is linearly separable or not is unsurprising either way. The location and the width of this “transition region” are the two main parameters that summarize the shape of the separability curve. In Section 6 I will show how to compute these quantities within a more general model that includes data structure.

## 4. Quantifying Linear Separability via Relative Entropy

In order to make a step further in the characterization of the linear separability of (rescaled) MNIST, we can consider its subsets. While there is only one subset with m=2000 points (focusing on the dichotomy “3”/“7”), and only one yes/no answer to the question of its linear separability, there are many subsets of size p<m, which can provide more detailed information. To quantify such information, let us formulate a more precise notion of surprise with respect to a model expressing prior expectation [39]. Let us again fix an empirical data set $Z_{m}={\left\{\left(\xi_{\mu },\sigma_{\mu }\right)\right\}}_{\mu =1,\ldots ,m}$ and fix p≤m. Now, consider the set $N_{p}$ of all subsets $\nu =\{\nu_{1},\ldots ,\nu_{p}\}$ of *p* indices $\nu_{i}\in \left\{1,\ldots ,m\right\}$, with $\nu_{i}\neq \nu_{j}$ for i≠j. Additionally, consider the set $\Sigma_{p}={\left\{-1,+1\right\}}^{p}$ of all dichotomies $\hat{\sigma }=\left\{{\hat{\sigma }}_{1},\ldots ,{\hat{\sigma }}_{p}\right\}$ of *p* elements. (I use curly braces for both sets and indexed families.) For each pair $\left(\nu \in N_{p},\hat{\sigma }\in \Sigma_{p}\right)$, we can construct the corresponding synthetic dataset

(3) $$Z\left(\nu ,\hat{\sigma }\right)={\left\{(\xi_{\nu_{i}},{\hat{\sigma }}_{i})\right\}}_{i=1,\ldots ,p};$$

similarly, for each $\nu \in N_{p}$, we can construct the corresponding subset $Z_{\mathrm{emp}}\left(\nu \right)$ of the empirical data set $Z_{m}$:

(4) $$Z_{\mathrm{emp}}\left(\nu \right)={\left\{(\xi_{\nu_{i}},\sigma_{\nu_{i}})\right\}}_{i=1,\ldots ,p}.$$

The main tool for defining the surprise will be probability distributions on a space $\Omega_{p}$, which is defined as the union of all synthetic data sets:

(5) $$\Omega_{p}=\underset{\left(\nu ,\hat{\sigma }\right)\in N_{p}\times \Sigma_{p}}{⋃}Z\left(\nu ,\hat{\sigma }\right).$$

The empirical space $\Omega_{p}^{\mathrm{emp}}\subseteq \Omega_{p}$ can be defined similarly:

(6) $$\Omega_{p}^{\mathrm{emp}}=\underset{\nu \in N_{p}}{⋃}Z_{\mathrm{emp}}\left(\nu \right).$$

Essentially, $\Omega_{p}^{\mathrm{emp}}$ contains all collections of *p* point/label pairs in the data set $Z_{m}$, while $\Omega_{p}$ contains all the collections of *p* point/label pairs where the *p* points are chosen among the ones in the data set and the labels are all possible $2^{p}$ combinations on those *p* points. Notice that $\Omega_{p}$ and $\Omega_{p}^{\mathrm{emp}}$ have different cardinalities: $|\Omega_{p}^{\mathrm{emp}}|=M_{p}$ and $|\Omega_{p}|=2^{p}M_{p}$, where Mp=mp is the number of subsets of size *p* in the data set.

Interpreted as a probability distribution on $\Omega_{p}$, the empirical data are uniform distributed on $\Omega_{p}^{\mathrm{emp}}$; likewise, the null model defined above induces, by conditioning on the points $\left\{\xi_{\mu }\right\}$, the uniform distribution on the whole $\Omega_{p}$. In general, not every data set in $\Omega_{p}$ (nor in $\Omega_{p}^{\mathrm{emp}}$) is linearly separable. Let us define the subsets for which this property holds:

(7) $$\begin{array}{cc} \; & {\hat{\Omega }}_{p}=\left\{Z\in \Omega_{p}:Z\;\mathrm{is}\;\mathrm{linearly}\;\mathrm{separable}\right\}\\ \; & {\hat{\Omega }}_{p}^{\mathrm{emp}}=\left\{Z\in \Omega_{p}^{\mathrm{emp}}:Z\;\mathrm{is}\;\mathrm{linearly}\;\mathrm{separable}\right\}. \end{array}$$

Let us call $Q_{p}$ and $Q_{p}^{\mathrm{emp}}$ the uniform probability distributions on ${\hat{\Omega }}_{p}$ and ${\hat{\Omega }}_{p}^{\mathrm{emp}}$, respectively. The Kullback–Leibler (KL) divergence $D_{\mathrm{KL}}\left(Q_{p}^{\mathrm{emp}}\;\left|\right|\;Q_{p}\right)$ from $Q_{p}$ to $Q_{p}^{\mathrm{emp}}$ (or relative entropy)

(8) $$D_{\mathrm{KL}}\left(Q_{p}^{\mathrm{emp}}\;\left|\right|\;Q_{p}\right)=\sum_{z\in \Omega_{p}}Q_{p}^{\mathrm{emp}}\log\frac{Q_{p}^{\mathrm{emp}}}{Q_{p}}$$

then measures the surprise carried by the data with respect to the prior belief regarding its linear separability expressed by $Q_{p}$. Because $Q_{p}$ and $Q_{p}^{\mathrm{emp}}$ are defined on sets ($\Omega_{p}$ and $\Omega_{p}^{\mathrm{emp}}$) of different cardinality, I define the (signed) surprise $S_{p}$ by subtracting the reference KL divergence between the uniform distributions on these spaces:

(9) $$\begin{array}{cc} S_{p} & =D_{\mathrm{KL}}\left(Q_{p}^{\mathrm{emp}}\;\left|\right|\;Q_{p}\right)-\log\left(\left|\Omega_{p}\right|/\left|\Omega_{p}^{\mathrm{emp}}\right|\right)\\ \; & =D_{\mathrm{KL}}\left(Q_{p}^{\mathrm{emp}}\;\left|\right|\;Q_{p}\right)-p\log2. \end{array}$$

Notice that the summand in the definition of KL divergence, Equation (8), is only nonzero for $z\in {\hat{\Omega }}_{p}^{\mathrm{emp}}$; one then obtains

(10) $$\begin{array}{cc} S_{p} & =\sum_{z\in {\hat{\Omega }}_{p}^{\mathrm{emp}}}\frac{1}{|{\hat{\Omega }}_{p}^{\mathrm{emp}}|}\log\frac{|{\hat{\Omega }}_{p}|}{|{\hat{\Omega }}_{p}^{\mathrm{emp}}|}-p\log2\\ \; & =\log\frac{|{\hat{\Omega }}_{p}|}{|{\hat{\Omega }}_{p}^{\mathrm{emp}}|}-p\log2\\ \; & =\log\frac{c_{n,p}M_{p}2^{p}}{|{\hat{\Omega }}_{p}^{\mathrm{emp}}|}-p\log2\\ \; & =\log c_{n,p}-\log c_{n,p}^{\mathrm{emp}}, \end{array}$$

where I have defined the empirical separability $c_{n,p}^{\mathrm{emp}}$ as the fraction of linearly separable subsets of size *p* in $Z_{m}$:

(11) $$c_{n,p}^{\mathrm{emp}}=\frac{|{\hat{\Omega }}_{p}^{\mathrm{emp}}|}{M_{p}}.$$

The signed surprise $S_{p}$ is positive (respectively negative) when the fraction of linearly separable subsets of size *p* is smaller (respectively larger) than expected in the null model.

### Separability in a Synthetic Data Set and in MNIST

The discussion above encourages the use of the empirical separability $c_{n,p}^{\mathrm{emp}}$ as a detailed description of the linear separability of a data set in an information theoretic framework. Despite being one of the simplest benchmark data sets used in machine learning, MNIST is already rather complex; its classes are known to have small intrinsic dimensions and varied geometries [15]. Therefore, before turning to MNIST, let us consider a simple controlled experiment, where the data are extracted from a simple one-parameter mixture distribution, defined, as follows. Let σ∈{−1,+1} be a Bernoulli random variable with parameter 1/2, which generates the labels. The data points $\xi \in R^{n}$ are extracted from a multivariate normal distribution with σ-dependent mean. The joint probability distribution of each point-label pair is

(12) $$P\left(\left\{\xi ,\sigma \right\}\right)=\frac{1}{2}f_{N\left(\mu (\sigma ),I\right)}\left(\xi \right),\;\mu \left(\sigma \right)=\left(\sigma \frac{\delta }{2},0,\ldots ,0\right),$$

where $f_{N(\mu ,I)}$ is the probability density function of the multivariate normal distribution with mean μ and identity covariance matrix. The parameter δ measures the distance between the two means: $\delta =∥\mu (\sigma =+1)-\mu (\sigma =-1)∥$. Figure 2 shows the empirical separability $c_{n,p}^{\mathrm{emp}}$, as a function of the size *p* of the subsets, for such a data set containing m=200 data points in n=20 dimensions. When δ=0, all of the data points are extracted from the same distribution, regardless of their labels: the data have no structure and the separability follows the null model, as in Equation (2). While δ increases, equally labelled points start to cluster, and the separability at any given p>n increases, as expected from the qualitative discussion in Section 2. It is interesting to note that the width of the transition region (Δp in Figure 1) is also an increasing function of δ. This dependence was not expected *a priori*; In Section 7, I will show that the theory of structured data presented below allows for explaining this behavior.

Let us now compute $c_{n,p}^{\mathrm{emp}}$ for the rescaled MNIST data set. Figure 2 shows the results of three numerical experiments, as compared with the null model prediction (2), and elicits four observations. (i) MNIST data are significantly more separable than the null model. For instance, the signed surprise, with respect to the null model, of the empirical dichotomies separating the digits “3” and “7” takes the values $S_{400}\approx -55$, $S_{500}\approx -100$, $S_{600}\approx -150$. (ii) Even within the same data set, different classifications can have different probabilities of separation; the dichotomy separating the digits “4” and “9” in rescaled MNIST is closer to the null model than the dichotomy of “3” and “7” (e.g., $S_{400}\approx -48$). (iii) Destroying the structure by random reshuffling of the labels makes the separability collapse onto that of the null model; the surprise $S_{p}$ in this case is, at most, of order $10^{-1}$ for all *p*. (iv) Similarly to what happens in the more controlled experiment with the synthetic data above, the separability curve of the “3”/“7” dichotomy, which has its transition point at a larger value of *p* than the “3”/“9” dichotomy, also has a wider transition region.

This analysis shows that, contrary to what appeared by looking solely at the whole data set, the dichotomies of rescaled MNIST are much more likely to be realized by a linear separator than random ones. In relation to the separability as a function of *p*, the null model has a single parameter, the dimension *n*. Is it possible to interpret the empirical curves as those of the null model with an effective dimension $n_{\mathrm{eff}}$? Increasing *n* has the effect of increasing proportionally the value $p_{c}$ because the storage capacity is fixed to $\alpha_{c}=2$. However, while fixing $n_{\mathrm{eff}}\approx 280$ indeed aligns the critical number of points $p_{c}$ with the empirical one, it yields a much smaller width of the transition region (Δp≈80 for the null model and Δp≈300 in the data). Furthermore, notice that the values of the surprise for the “3”-vs.-“7” and “4”-vs.-“9” experiments are not very different. The reason is the ingenuousness of the null model, which hardly captures the properties of the empirical sets, and whose term $c_{n,p}$ therefore dominates in $S_{p}$. These observations, together with the motivations that are discussed above, are a spur for the definition of a more nuanced and versatile model of the separability of structured data.

## 5. Parameterized Model of Structured Data

Fixing a model of data structure in this context means fixing a generative model of data. Here, I use the model first introduced in [28]. This should not be considered to be a realistic model of real data sets. It is useful as an effective or phenomenological parameterization of data structure. It has two main advantages: (i) it allows the analytical computation, within a mean field approximation, of the probability of separation $c_{n,p}$; and, (ii) it naturally points out the relevant geometric-probabilistic parameters that control the linear separability.

The model is expressed in the form of constraints between the points and the labels. The synthetic data set is constructed as a collection of *q* “multiplets”, i.e., subsets of *k* points $\left\{\xi_{\mu }^{1},\ldots ,\xi_{\mu }^{k}\right\}$ with prescribed geometric relations between them, and such that the labels are constant within each multiplet:

(13) Zq=ξ11,σ1,⋯,ξ1k,σ1,{ξ21,σ2,⋯,ξ2k,σ2,{ξ21,σ2,⋮{ξq1,σq,⋯,ξqk,σq.

The total number of point/label pairs is p=qk. Observe that, if one considers the set of all points $X=\{\xi_{\mu }^{i}\}$, not every dichotomy of *X* is admitted by the parameterization of $Z_{q}$ in Equation (13). If a dichotomy assigns different labels to two elements of the same multiplet, it cannot be written in this form. The dichotomies that agree with the parameterization of Equation (13) are termed as admissible.

The relations between the points $\xi_{\mu }^{i}$ within each multiplet can be fixed, for instance, by prescribing that the k(k−1)/2 overlaps $\rho_{i,j}=\xi_{\mu }^{i}\cdot \xi_{\mu }^{j}$ be fixed and independent of μ (remember that $|\xi_{\mu }^{i}|=1$). The statistical ensemble for $Z_{q}$, as specified by the probability density $dp(Z_{q})$, is chosen in accordance with the maximum entropy principle: it is the uniform probability distribution on the points and the labels independently, given the constraints:

(14) $$dp\left(Z_{q}\right)=\frac{1}{Z\left(n,q,\{\rho_{i,j}\}\right)}\prod_{\begin{array}{c} \mu =1,\ldots ,q\\ i=1,\ldots ,k \end{array}}\;\;d\xi_{\mu }^{i}\;\delta \left(\left|\xi_{\mu }^{i}\right|-1\right)\;\;\prod_{a>b=1,\ldots ,k}\;\;\delta \left(\rho_{a,b}-\xi_{\mu }^{a}\cdot \xi_{\mu }^{b}\right),$$

where $Z\left(n,q,\{\rho_{i,j}\}\right)$ is the partition function, fixed by the normalization condition

(15) $$\sum_{\left\{\sigma_{\mu }\right\}}\int_{R^{nqk}}\;dp\left(Z_{q}\right)=1.$$

The null (unstructured) model of Section 3 is recovered in this parameterization in two different limits. First, if k=1 each multiplet is composed of a single point, and no contraints are imposed other than the normalization. Second, for any *k*, if all overlaps are fixed to 1, then all points in each overlap coincide, $\xi_{\mu }^{1}=\xi_{\mu }^{2}=\cdots =\xi_{\mu }^{k}$, and the model is equivalent to the null model with p=q.

The theory that will be described below depends on a natural set of parameters $\psi_{m}$, with m=2,…,k. These quantities are conditional probabilities of geometric events that are related to single multiplets. They characterize the properties of the multiplets that are relevant for the linear separability of the whole set. Consider a multiplet $X=\{\xi^{1},\ldots ,\xi^{k}\}$. $\psi_{m}$ is a measure of the likelihood that a subset $X^{\prime }\subseteq X$ of m≤k points is classified coherently by a random weight vector. More precisely, $\psi_{m}$ is the probability that the scalar product w·ξ has the same sign for all $\xi \in X^{\prime }$, being conditioned on the event that w·ξ has the same sign for all $\xi \in X\backslash \{\xi_{🟉}\}$. This probability is computed in the ensemble where the vector *w* is uniformly distributed on the unit sphere $S^{n-1}$, $X^{\prime }$ is uniformly distributed on the subsets of *X* of *m* points, and $\xi_{🟉}$ is uniformly distributed on the elements of $X^{\prime }$. This is coherent with the mean field nature of the combinatorial theory, which assumes uniformly distributed and uncorrelated quantities (see below).

In a few cases, $\psi_{m}$ can be computed explicitly. For instance, for a doublet $\left\{\xi ,\bar{\xi }\right\}$ at fixed overlap $\rho =\xi \cdot \bar{\xi }$,

(16) $$\psi_{2}\left(\rho \right)=\frac{2}{\pi }\arctan\sqrt{\frac{1+\rho }{1-\rho }}.$$

This is the probability that a random hyperplane does not intersect the segment that connects two points at overlap ρ. It is an increasing function of ρ, from $\psi_{2}\left(-1\right)=0$ to $\psi_{2}\left(1\right)=1$. If k>2, then the quantity that enters the equations will be the mean of $\psi_{2}\left(\rho \right)$ over all the pairs in the multiplet. It can be shown that $\psi_{m}$, as a function of the overlaps $\rho_{i,j}$, does not explicitly depend on the dimensionality *n* [28]; this property greatly simplifies the analytical computations.

In summary, the parameters of the model are the following: the dimensionality *n*, the multiplicity *k*, and the k−2 probabilities $\psi_{m}$. Actually, only two special combinations of the parameters $\psi_{m}$ emerge as relevant from the theory that is presented in the next sections:

(17) $$\begin{array}{cc} \Psi_{1} & =\sum_{r=2}^{k}\psi_{r}, \end{array}$$

(18) $$\begin{array}{cc} \Psi_{2} & =\sum_{r=2}^{k}\sum_{l=2}^{r-1}\psi_{r}\psi_{l}. \end{array}$$

I will call them structure parameters. Other functions of the probabilities $\psi_{m}$ are relevant for other purposes, for instance, when considering the large-*p* asymptotics of $c_{n,p}$, which relates to the generalization properties of the linear separator [32].

## 6. Combinatorial Computation of the Separability for Structured Data

Cover popularized a powerful combinatorial technique to compute the number of linearly realizable dichotomies in an old and highly cited paper [38]. Despite its appeal, the combinatorial approach (while certainly not extraneous to contemporary statistical physics, both theoretical and applied [40,41,42,43]) remained somewhat confined to very few papers in discrete mathematics, and it was only very recently extended to more modern questions, when it was used to obtain an equation for $C_{n,q}$, the number of admissible dichotomies of *q* multiplets, for structured data of the type that is defined in the previous section. Ref. [28] first presented the arguments and computations leading to this equation. To make this article as self-contained as possible, I repeat most of the derivation here.

### 6.1. Exact Approach for Unstructured Data (k = 1 Points per Multiplet)

First, I recall the classic computation for unstructured data (k=1 in our notation). The idea is to write a recurrence relation for the number of linearly realizable dichotomies $C_{n,p}$ and, consequently, for the probability $c_{n,p}$, by considering the addition of the (p+1)th element $\xi_{p+1}$ to the set $X_{p}=\left\{\xi_{1},\ldots ,\xi_{p}\right\}$ that was composed of the first *p* elements.

Consider one of the dichotomies of $X_{p}$, let us call it $\phi_{p}$; how many linearly realizable dichotomies of $X_{p+1}=\left\{\xi_{1},\ldots ,\xi_{p},\xi_{p+1}\right\}$ agree with $\phi_{p}$ (i.e., take the same values) on the points of $X_{p}$? When the point $\xi_{p+1}$ is added to the set, two different things can happen: (i) $\mathrm{sgn}(w\cdot \xi_{p+1})$ is the same for all possible weight vectors *w* that realize $\phi_{p}$; and, (ii) there is at least one weight vector $\hat{w}$ realizing $\phi_{p}$, such that $\hat{w}\cdot \xi_{p+1}=0$. These two cases lead to different contributions to $C_{n,p+1}$. In the first case, there is only one dichotomy of $X_{p+1}$ agreeing with $\phi_{p}$, as the value that is assigned to $\xi_{p+1}$ is fixed. In the second case, the value that is assigned to $\xi_{p+1}$ can be either +1 or −1; therefore, the number of dichotomies of $X_{p+1}$ agreeing with $\phi_{p}$ is 2.

Let us call $M_{n,p}$ the number of those dichotomies, among the $C_{n,p}$ dichotomies of $X_{p}$, such that (ii) holds for the new point; the number of those satisfying (i) will be $C_{n,p}-M_{n,p}$. The reasoning above then leads to $C_{n,p+1}=\left(C_{n,p}-M_{n,p}\right)+2M_{n,p}=C_{n,p}+M_{n,p}$. Here lies the keystone that allows for the closure of the recurrence equation: $M_{n,p}$ is the number of dichotomies conditioned to satisfy a linear constraint; therefore, it is equal to the number of dichotomies, of the same number of points *p*, in n−1 dimensions: $M_{n,p}=C_{n-1,p}$. Finally, the recurrence relation is $C_{n,p+1}=C_{n,p}+C_{n-1,p}$, which translates into the following equation for the probability $c_{n,p}$:

(19) $$c_{n,p+1}=\frac{1}{2}\left(c_{n,p}+c_{n-1,p}\right).$$

The boundary conditions of the recurrence (19) are

(20) $$\begin{array}{cc} \; & c_{n>0,1}=1,\\ \; & c_{n\leq 0,p}=0\;\left[\Rightarrow c_{1,p}=2^{1-p}\right], \end{array}$$

which come from the conditions $C_{1,p>0}=2$ (there are only two normalized weight vectors in one dimension) and $C_{n>0,1}=2$ (there is always a weight vector *w*, such that ±w·ξ=±1). The solution of Equation (19) is Equation (2), as can be checked directly. However, the more complicated equations that are satisfied by the probabilities for structured data are not as easily solvable. For this reason, in Section 7, below, I will show a method to compute useful quantities that are related to the shape of $c_{n,p}$ directly from the recurrence relations, with no need for a closed solution.

### 6.2. Mean-Field Approach for Pairs of Points (k = 2 Points per Multiplet)

The simplest non-trivial extension of Cover’s computation to structured data is k=2. From here on I will use ${\hat{c}}_{n,q}$ and ${\hat{C}}_{n,q}$ to denote the fraction and number of linearly realizable admissible dichotomies of *q* multiplets because the symbols $c_{n,p}$ and $C_{n,p}$ were reserved to denote the fraction and number of linearly realizable dichotomies of *p* points.

Notice that all the quantities appearing above are notated with no explicit dependence on the points ξ. This is because the unstructured case enjoys a strong universality property (as proved in [38]): $C_{n,p}$ is independent of the points of $X_{p}$, as long as they are in a general position. Such generality breaks down for structured data. In this case, the recurrence equations that will be obtained are not valid for all sets $X_{p}$; rather, they are satisfied by the ensemble averages of ${\hat{C}}_{n,q}$ and ${\hat{c}}_{n,q}$, in the spirit of the mean-field approximation of statistical physics.

The set of points is now $X_{q}\cup {\bar{X}}_{q}$, where $X_{q}$ is a set of *q* points $\left\{\xi_{1},\ldots ,\xi_{q}\right\}$ and ${\bar{X}}_{q}$ is a set of partners $\left\{{\bar{\xi }}_{1},\ldots ,{\bar{\xi }}_{q}\right\}$, where $\xi_{\mu }\cdot {\bar{\xi }}_{\mu }=\rho$ for all μ=1,…,q (remember that all of the points are on the unit sphere). Consider the addition of the points $\xi_{q+1}$ and ${\bar{\xi }}_{q+1}$ to $X_{q}$ and ${\bar{X}}_{q}$, respectively. By repeating the reasoning described above for k=1 with respect to the point ${\bar{\xi }}_{q+1}$, one finds a formula for the number $Q_{n,q}$ of dichotomies of the set $\left\{\xi_{1},{\bar{\xi }}_{1},\ldots ,\xi_{q},{\bar{\xi }}_{q},{\bar{\xi }}_{q+1}\right\}$ that are admissible on the first *q* pairs (and are unconstrained on ${\bar{\xi }}_{q+1}$): $Q_{n,q}={\hat{C}}_{n,q}+{\hat{C}}_{n-1,q}$. These dichotomies can be separated into two classes, similarly to the two cases (i) and (ii) above: those that can be realized by a weight vector orthogonal to $\xi_{q+1}$ (let us denote their number by $R_{n,q}$) and those that cannot (their number is then $Q_{n,q}-R_{n,q}$). For each dichotomy ϕ of the first class, there exists one and only one admissible dichotomy of the full set $X_{q+1}\cup {\bar{X}}_{q+1}$ that agrees with ϕ and can be realized linearly. In fact, thanks to the orthogonality constraint, there is always, among the weight vectors realizing ϕ, one vector *w*, such that

(21) $$\mathrm{sgn}\left(w\cdot \xi_{q+1}\right)=\phi \left({\bar{\xi }}_{q+1}\right),$$

thus satisfying the admissibility condition on the pair $\left\{\xi_{q+1},{\bar{\xi }}_{q+1}\right\}$. The remaining $Q_{n,q}-R_{n,q}$ dichotomies do not allow this freedom. How many of them are realized by weight vectors *w*, such that the admissibility condition (21) is satisfied can be estimated at the mean field level by the probability that, given a random weight vector *w* chosen uniformly on the unit sphere, the scalar products $w\cdot \xi_{q+1}$ and $w\cdot {\bar{\xi }}_{q+1}$ have the same sign. This probability does not depend on the actual points, but only on their overlap ρ, and it is exactly the quantity $\psi_{2}\left(\rho \right)$ that is defined in the previous section, Equation (16). I will denote it by $\psi_{2}$ in the following, with the dependence on ρ being understood.

The foregoing argument brings the following equation:

(22) $${\hat{C}}_{n,q+1}=R_{n,q}+\psi_{2}\left({\hat{C}}_{n,q}+{\hat{C}}_{n-1,q}-R_{n,q}\right)$$

Similarly to what happens in the unstructured case, the unknown term $R_{n,q}$ can be expressed in terms of variables ${\hat{C}}_{•,q}$ by considering the same problem in a lower dimension. In fact, remember that $Q_{n,q}$ above was computed by applying Cover’s argument for k=1, because it counts how the number of dichotomies is affected when the single point ${\bar{\xi }}_{q+1}$ is added to the set. $R_{n,q}$ must be computed in the same way, since it, again, counts the number of dichotomies that are admissible on the first *q* pairs and free on ${\bar{\xi }}_{q+1}$. However, these dichotomies must satisfy the additional linear constraint $w\cdot \xi_{q+1}=0$; therefore, the whole argument must be applied in n−1 dimensions. This leads to

(23) $$R_{n,q}={\hat{C}}_{n-1,q}+{\hat{C}}_{n-2,q}.$$

Finally, substituting this expression of $R_{n,q}$ into Equation (22) yields

(24) $${\hat{C}}_{n,q+1}=\psi_{2}{\hat{C}}_{n,q}+{\hat{C}}_{n-1,q}+\left(1-\psi_{2}\right){\hat{C}}_{n-2,q}.$$

As above, this translates to a similar equation for the probability ${\hat{c}}_{n,q}$:

(25) $${\hat{c}}_{n,q+1}=\frac{\psi_{2}}{2}{\hat{c}}_{n,q}+\frac{1}{2}{\hat{c}}_{n-1,q}+\frac{1-\psi_{2}}{2}{\hat{c}}_{n-2,q}.$$

The boundary conditions of this recurrence are slightly different than for k=1. They are discussed in the Appendix A, together with those for the general case.

### 6.3. General Case Parameterized by k

It is possible to extend the method that is described above to all *k*. I will only sketch the derivation; the details can be found in [28]. Just as the case k=2 can be treated by making use of the recurrence formula for k=1, the idea here is to construct the case *k* recursively by using the formula (yet to be found) for k−1, therefore obtaining a recurrence relation in *k* as well as in *n* and *q*. To this aim, the (q+1)th multiplet $\left\{\xi_{q+1}^{1},\ldots ,\xi_{q+1}^{k}\right\}$ is split into the two subsets $\left\{\xi_{q+1}^{1}\right\}$ and ${\bar{\xi }}_{q+1}=\left\{\xi_{q+1}^{2},\ldots ,\xi_{q+1}^{k}\right\}$. The formula for k−1 allows for applying the argument to the set ${\bar{\xi }}_{q+1}$, thus obtaining the number $Q_{n,q}$ of dichotomies of the set $X_{q}\backslash \left\{\xi_{q+1}^{1}\right\}$ that are admissible on the first *q* complete multiplets and are admissible on the (q+1)th incomplete multiplet ${\bar{\xi }}_{q+1}$. More formally, $Q_{n,q}$ is the number of linearly realizable dichotomies ϕ, such that

(26) $$\begin{array}{cc} \; & \phi \left(\xi_{\mu }^{i}\right)=\phi \left(\xi_{\mu }^{j}\right)\;i,j=1,\ldots ,k;\;\mu =1,\ldots ,q\\ \; & \phi \left(\xi_{q+1}^{i}\right)=\phi \left(\xi_{q+1}^{j}\right)\;i,j=2,\ldots ,k. \end{array}$$

Now the argument goes exactly as for the case k=2: some of these $Q_{n,q}$ dichotomies (their number being $R_{n,q}$) can be realized by a weight vector orthogonal to the point $\xi_{q+1}^{1}$; therefore, each of them contributes a single admissible dichotomy of the whole set $X_{q+1}$; the remaining $Q_{n,q}-R_{n,q}$ contribute with probability $\psi_{k}$. Again, $R_{n,q}$ can be expressed by applying the same argument in n−1 dimensions.

Finally, one finds that the probability ${\hat{c}}_{n,q}$ satisfies a recurrence equation in *n* and *q*:

(27) $${\hat{c}}_{n,q+1}=\sum_{l=0}^{k}\theta_{l}^{k}c_{n-l,q},$$

where the coefficients $\theta_{l}^{k}$ are constants (independent of *n* and *q*) satisfying a recurrence equation in *k* and *l*:

(28) $$\theta_{l}^{k}=\psi_{k}\theta_{l}^{k-1}+\left(1-\psi_{k}\right)\theta_{l-1}^{k-1}.$$

The boundary conditions for Equation (28) are

(29) $$\begin{array}{cc} \; & \theta_{0}^{1}=\theta_{1}^{1}=\frac{1}{2}\\ \; & \theta_{l<0}^{k}=\theta_{l>k}^{k}=0; \end{array}$$

the conditions at k=1 are those that reproduce Equation (19).

## 7. Computation of Compact Metrics of Linear Separability

The model of data structure leading to the foregoing equations is very detailed, in that it allows for the independent specification of a large number of parameters. However, the influence of each parameter on the separability ${\hat{c}}_{n,q}$ is not equal, with some combinations of parameters being more relevant than others. In this section, I compute two main descriptors of the shape of ${\hat{c}}_{n,q}$ as a function of *q* at *n* fixed: the transition point $p_{c}$ (equivalently, the capacity $\alpha_{c}$) and the width Δp of the transition region; they are defined more precisely below. We will see that only the structure parameters $\Psi_{1}$ and $\Psi_{2}$, the special combinations defined in Section 5, are needed to fix $p_{c}$ and Δp.

### 7.1. Diagonalization of the Recurrence Relation

Notice that, while the quantity ${\hat{c}}_{n,q}$ that is given by the theory is expressed as a function of the number of multiplets *q*, the definition of separability that is discussed in Section 5 is given in terms of the number of points p=kq. This is not really a problem in the thermodynamic limit

(30) $$\begin{array}{cc} \; & n\to \infty \\ \; & p\to \infty \\ \; & \alpha =\frac{p}{n}\;\;\mathrm{fixed}\\ \; & k\;\;\mathrm{fixed}, \end{array}$$

whereby the separability is expressed as a function of the load α. In the following, I will define the location $q_{c}$ and the width Δq of the transition region in the parameterization by the number of multiplets *q*; the corresponding quantities that are parameterized by *p* are obtained by rescaling:

(31) $$p_{c}=kq_{c},\;\Delta p=k\Delta q.$$

Let us consider the discrete derivative of ${\hat{c}}_{n,q}$ with respect to *n*:

(32) $$\gamma_{n,q}=\Delta_{n}{\hat{c}}_{n,q}\equiv {\hat{c}}_{n+1,q}-{\hat{c}}_{n,q}.$$

As will be clear momentarily, working with $\gamma_{n,q}$ is convenient because it is normalized, as I will prove below. $\gamma_{n,q}$ satisfies the same recurrence relation as ${\hat{c}}_{n,q}$:

(33) $$\gamma_{n,q+1}=\sum_{l=0}^{k}\theta_{l}^{k}\gamma_{n-l,q}.$$

The boundary conditions, in accordance with (20), are

(34) $$\begin{array}{cc} \; & \gamma_{n,1}=\delta_{n,0},\\ \; & \gamma_{n<0,q}=0. \end{array}$$

The right hand side of Equation (33) has the form of a discrete convolution between $\theta_{•}^{k}$ and $\gamma_{•,q}$:

(35) $$c_{•,q+1}=\theta_{•}^{k}*c_{•,q}.$$

The convolution is diagonalized in Fourier space, by defining the characteristic functions

(36) $$\begin{array}{cc} {\tilde{\gamma }}_{q}\left(t\right)= & \sum_{n=0}^{\infty }\gamma_{n,q}e^{int}, \end{array}$$

(37) $$\begin{array}{cc} {\tilde{\theta }}_{k}\left(t\right)= & \sum_{n=0}^{\infty }\theta_{n}^{k}e^{int}. \end{array}$$

Multiplying both sides of Equation (35) by $e^{int}$ and summing over *n* yields

(38) $${\tilde{\gamma }}_{q+1}\left(t\right)={\tilde{\theta }}_{k}\left(t\right)\;{\tilde{\gamma }}_{q}\left(t\right).$$

From the definition (36) and boundary conditions (34), one gets ${\tilde{\gamma }}_{1}\left(t\right)=1$; hence, the solution of the recurrence equation is

(39) $${\tilde{\gamma }}_{q}\left(t\right)={\left[{\tilde{\theta }}_{k}\left(t\right)\right]}^{q-1}.$$

### 7.2. Defining the Location and Width of the Transition Region

As mentioned above, $\gamma_{n,q}$ is normalized, which means that

(40) $$\sum_{n=0}^{\infty }\gamma_{n,q}=1,$$

or, equivalently, ${\tilde{\gamma }}_{q}\left(0\right)=1$. To prove this, it suffices to show that ${\tilde{\theta }}_{k}\left(0\right)=1$, i.e., that $\theta_{n}^{k}$ is normalized. Summing both sides of Equation (28) in *l* from 0 to *∞* shows that ${\tilde{\theta }}_{k}\left(0\right)$ is constant in *k*, therefore

(41) $${\tilde{\theta }}_{k}\left(0\right)={\tilde{\theta }}_{1}\left(0\right)=1,$$

as can be computed from the boundary conditions (29).

Because it is normalized, $\gamma_{•,q}$ can be interpreted as a probability distribution, whose cumulative distribution function is ${\hat{c}}_{•,q}$. The *a*th moment of the distribution is

(42) $$\begin{array}{cc} {\langle n^{a}\rangle }_{q} & =\sum_{n=0}^{\infty }n^{a}\gamma_{n,q}\\ \; & =i^{-a}{\left.\frac{d^{a}}{dt^{a}}{\tilde{\gamma }}_{q}\left(t\right)\right|}_{t=0}. \end{array}$$

The same holds for $\theta_{•}^{k}$, whose moments ${\langle \theta^{a}\rangle }_{k}$ can be obtained from its characteristic function ${\tilde{\theta }}_{k}\left(t\right)$. Let us focus on the mean $\mu_{q}$ and the variance $\sigma_{q}$,

(43) $$\mu_{q}={\langle n\rangle }_{q},\;\sigma_{q}^{2}={\langle n^{2}\rangle }_{q}-{\langle n\rangle }_{q}^{2}.$$

Equation (39) allows for expressing these quantities in terms of the mean $\mu_{\theta }={\langle \theta \rangle }_{k}$ and variance $\sigma_{\theta }^{2}={\langle \theta^{2}\rangle }_{k}-{\langle \theta \rangle }_{k}^{2}$ of $\theta_{•}^{k}$:

(44) $$\mu_{q}=\left(q-1\right)\mu_{\theta },\;\sigma_{q}^{2}=\left(q-1\right)\sigma_{\theta }^{2},$$

as can be checked by using Equation (42).

We can now define the two main descriptors, $q_{c}$ and Δq, which summarize the separability as a function of *q*:

(45) $$\begin{array}{cc} q_{c}:\; & \mu_{q_{c}}=n, \end{array}$$

(46) $$\begin{array}{cc} q_{\pm }:\; & \mu_{q_{\pm }}\mp \sigma_{q_{\pm }}=n, \end{array}$$

(47) $$\begin{array}{cc} \Delta q:\; & \Delta q=q_{+}-q_{-}. \end{array}$$

### 7.3. Expression in Terms of the Structure Parameters

To compute these quantities, all we need is $\mu_{\theta }$ and $\sigma_{\theta }$, or ${\langle \theta \rangle }_{k}$ and ${\langle \theta^{2}\rangle }_{k}$. Solving Equation (45) for $q_{c}$ gives

(48) $$q_{c}=n\mu_{\theta }^{-1}+1.$$

Solving Equations (46) and (47) for Δq gives

(49) $$\Delta q=\frac{1}{\mu_{\theta }^{2}}\sqrt{\sigma_{\theta }^{2}\left(\sigma_{\theta }^{2}+4\mu_{\theta }n\right)}.$$

The corresponding expressions to leading order in *n* are the following

(50) $$\begin{array}{cc} q_{c} & =n\mu_{\theta }^{-1}+O\left(1\right),\\ \Delta q & =2\sigma_{\theta }\mu_{\theta }^{-3/2}n^{1/2}+O\left(n^{-1/2}\right). \end{array}$$

The moments of $\theta_{•}^{k}$ satisfy the following equation, which can be obtained by multiplying both sides of Equation (28) by $l^{a}$ and summing over *l*:

(51) θak=ψkθak−1+1−ψk∑l=0∞(l+1)aθlk−1=θak−1+1−ψk∑s=0a−1asθsk−1.

The boundary conditions are ${\langle \theta^{0}\rangle }_{k}=1$ (computed above) and ${\langle \theta^{a}\rangle }_{1}=1/2$, as given by Equation (29). In particular, for a=1, we obtain

(52) $${\langle \theta \rangle }_{k}={\langle \theta \rangle }_{k-1}+\left(1-\psi_{k}\right),$$

whose solution is

(53) $${\langle \theta \rangle }_{k}=k-\frac{1}{2}-\Psi_{1},$$

where the structure parameter $\Psi_{1}$, as defined in Equation (17), implicitly depends on *k*. For a=2, the recurrence Equation (51) becomes

(54) $${\langle \theta^{2}\rangle }_{k}={\langle \theta^{2}\rangle }_{k-1}+\left(1-\psi_{k}\right)\left(2{\langle \theta \rangle }_{k-1}+1\right).$$

By substituting ${\langle \theta \rangle }_{k-1}$ given by Equation (53) and solving the recurrence we obtain, after some algebra,

(55) $${\langle \theta^{2}\rangle }_{k}=k^{2}-k+\frac{1}{2}-2\left(k-1\right)\Psi_{1}+2\Psi_{2},$$

where $\Psi_{2}$ is the second structure parameter that is defined in Equation (18). Finally, by combining the leading order expansions (50) and the moments (53) and (55), and by rescaling, as in Equation (31), we have the following explicit expressions for the two main metrics of separability as functions of the multiplicity *k* and the structure parameters $\Psi_{1}$ and $\Psi_{2}$:

(56) $$\begin{array}{cc} \; & \frac{p_{c}}{n}={\left(1-\frac{1}{2k}-\frac{\Psi_{1}}{k}\right)}^{-1}, \end{array}$$

(57) $$\begin{array}{cc} \; & \frac{\Delta p}{\sqrt{n}}=2k{\left(k-\frac{1}{2}-\Psi_{1}\right)}^{-\frac{3}{2}}{\left(\frac{1}{4}+\Psi_{1}-\Psi_{1}^{2}+2\Psi_{2}\right)}^{\frac{1}{2}}. \end{array}$$

For data that are structured as pairs of points, k=2, Equation (56) gives the storage capacity of an ensemble of segments; this special result was first obtained, by means of replica calculations, in [44], and it was then rediscovered in other contexts in [8,45].

### 7.4. Dependence on the Structure Parameters and Scaling

The two structure parameters $\Psi_{1}$ and $\Psi_{2}$, which control the two main metrics of linear separability, belong to *k*-dependent ranges:

(58) $$\Psi_{1}\in \left[0,k-1\right],\;\Psi_{2}\in \left[0,\left(k-1\right)\left(k-2\right)/2\right].$$

The two quantities are not independent, since they are constructed from the same set of k−1 quantities $\psi_{m}\in \left[0,1\right]$. When conditioned on a fixed value of $\Psi_{1}$, $\Psi_{2}$ has a lower bound $\Psi_{2}^{-}$ and an upper bound $\Psi_{2}^{+}$ that can be computed by considering the two following extreme cases. First, the supremum of $\Psi_{2}$ is realized in the maximum entropy case, where the value of $\Psi_{1}$ is uniformly distributed among the $\psi_{m}$. Second, the infimum of $\Psi_{2}$ corresponds to the minimum entropy case, where $\Psi_{1}$ is distributed on the fewest possible $\psi_{m}$’s. Explicitly,

(59) $$\begin{array}{cc} \sup: & \;\left\{\psi_{m}\right\}=\left\{\frac{\Psi_{1}}{k-1},\cdots ,\frac{\Psi_{1}}{k-1}\right\}, \end{array}$$

(60) $$\begin{array}{cc} \inf: & \;\left\{\psi_{m}\right\}=\left\{\;\underset{\left\lfloor \Psi_{1}\right\rfloor }{\underset{︸}{1,\cdots ,1}}\;,\Psi_{1}-\left\lfloor \Psi_{1}\right\rfloor ,0,\cdots ,0\right\}. \end{array}$$

The definition of $\Psi_{2}$, Equation (18), can be rewritten, as follows:

(61) $$\Psi_{2}=\frac{1}{2}\Psi_{1}^{2}-\frac{1}{2}\sum_{m=2}^{k}\psi_{m}^{2}.$$

Substituting (59) and (60) into (61), we obtain

(62) $$\begin{array}{cc} \Psi_{2}^{+} & =\sup\Psi_{2}=\frac{\Psi_{1}^{2}}{2}\left(1-\frac{1}{k-1}\right), \end{array}$$

(63) $$\begin{array}{cc} \Psi_{2}^{-} & =\inf\Psi_{2}=\Psi_{1}\left\lfloor \Psi_{1}\right\rfloor -\frac{1}{2}\left({\left\lfloor \Psi_{1}\right\rfloor }^{2}+\left\lfloor \Psi_{1}\right\rfloor \right). \end{array}$$

**Figure 3:**
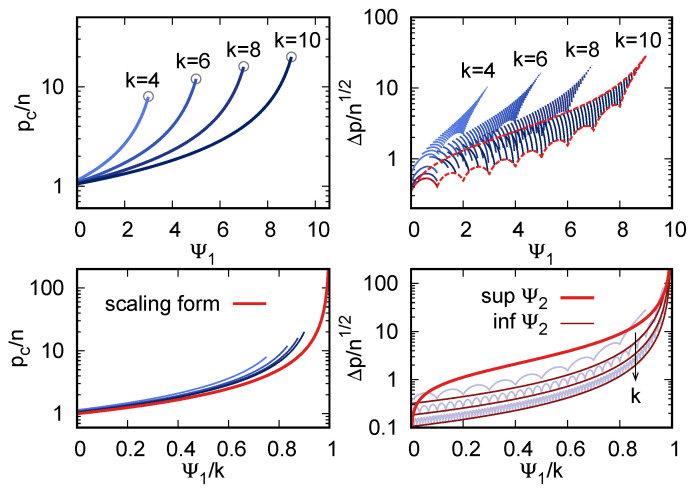
(**Top left**) The dependence of the rescaled location $p_{c}/n$ of the transition region (*y* axis) on the structure parameter $\Psi_{1}$ (*x* axis), for a few values of the multiplicity *k*. Circles pinpoint the tips of the curves, which correspond to the unstructured case, where $\Psi_{1}=k-1$ (i.e., $\psi_{m}=1$ for all *m*) and $p_{c}=2kn$. (**Top right**) The rescaled width $\Delta p/\sqrt{n}$ of the transition region (*y* axis). Segments correspond to 50 fixed values of $\Psi_{2}$, which were equally spaced in [0,(k−1)(k−2)/2]; their range in $\Psi_{1}$ (*x* axis) is obtained by inverting the relations (62) and (63). The dashed red lines are the upper and lower bounds of $\Delta p/\sqrt{n}$, obtained by substituting (62) and (63) into (57). (**Bottom left**) The large-*k* scaling form (red line) of $p_{c}/n$ (*y* axis) as a function of the rescaled parameter $\Psi_{1}/k$ (*x* axis); the blue lines are the same as in the top left panel. (**Bottom right**) The large-*k* behavior of the upper (thick red line) and lower (thin red and grey lines) bounds $\Delta p^{\pm }/\sqrt{n}$ (*y* axis) as functions of $\Psi_{1}/k$ (*x* axis). Grey lines are the tight lower bounds as in the top right panel and thin red lines are the simpler bound Equation (66); different lower bounds correspond to k=10, 30, 90.

Figure 3 shows the location of the transition, $p_{c}$, and the width of the region, Δp, as functions of $\Psi_{1}$ and $\Psi_{2}$ for a few values of *k*. Notice that the range of Δp at fixed *k* and $\Psi_{1}$ is itself bounded because of the limited range $\left[\Psi_{2}^{-},\Psi_{2}^{+}\right]$ of $\Psi_{2}$.

There is an interesting observation to be made on a semi-quantitative level. At fixed *k* and *n*, $p_{c}$ is an increasing function of $\Psi_{1}$. The width Δp depends on both structure parameters, but, since the range of $\Psi_{2}$ at fixed $\Psi_{1}$ is so limited, one expects that, in practice, Δp will be approximately an increasing function of $\Psi_{1}$. Therefore, Δp will be, in most cases, an increasing function of $p_{c}$. This is exactly the phenomenology that is observed in Figure 2, in both the synthetic data and MNIST.

The rescaled location of the transition $p_{c}/n$, Equation (56), does not depend on $\Psi_{2}$, and it depends on $\Psi_{1}$ only through the rescaled value $\Psi_{1}/k$. For large *k*, it takes the scaling form

(64) $$\frac{p_{c}}{n}∼f_{p_{c}}\left(\frac{\Psi_{1}}{k}\right),\;f_{p_{c}}\left(x\right)=\frac{1}{1-x}.$$

The width Δp, on the contrary, depends on both $\Psi_{1}$ and $\Psi_{2}$. Because it is a monotonically increasing function of $\Psi_{2}$, its upper bound $\Delta p^{+}$ and lower bound $\Delta p^{-}$ at fixed $\Psi_{1}$ can be obtained by substituting (62) and (63) in Equation (57). Expressing $\Delta p^{+}$ again as a function of the rescaled parameter $\Psi_{1}/k$, and only keeping the leading term in k→∞, one obtains the scaling form

(65) $$\frac{\Delta p^{+}}{\sqrt{n}}∼f_{\Delta p^{+}}\left(\frac{\Psi_{1}}{k}\right),\;f_{\Delta p^{+}}\left(x\right)=2\frac{\sqrt{x}}{1-x}.$$

Doing the same for $\Delta p^{-}$ yields a complicated function, which is plotted in Figure 3. A simpler expression for the bound can be obtained by observing that $\Psi_{2}^{-}\geq \left(\Psi_{1}^{2}-\Psi_{1}\right)/2$; using this more regular bound yields, at leading order in *k*,

(66) $$\frac{\Delta p^{-}}{\sqrt{n}}∼k^{-\frac{1}{2}}f_{\Delta p^{-}}\left(\frac{\Psi_{1}}{k}\right),\;f_{\Delta p^{-}}\left(x\right)={\left(1-x\right)}^{-\frac{3}{2}}.$$

Figure 3 shows the large-*k* scaling behavior of $p_{c}$, $\Delta p^{+}$, and $\Delta p^{-}$.

The two metrics are insensitive on most of the microscopic parameters of the theory, and they only depend on the two structure parameters, as shown analytically above. In addition, they display a large degree of robustness, even as functions of $\Psi_{1}$ and $\Psi_{2}$: measuring $p_{c}/n$ from the data fixes (up to corrections in *k*) the quantity $\Psi_{1}/k$, which, in turn, significantly narrows down the range of values that are attainable by Δp, the more so the smaller is *k*.

## 8. Discussion

The discussion above focused on the quantification of linear separability within a model that encodes simple relations between data points and their labels, in the form of constraints. Such a model has the advantage of being analytically tractable and allows the explicit expression of $p_{c}$ and Δp in terms of model parameters. Moreover, the parameters appearing in the theory have direct interpretations as probabilities of geometric events, thus suggesting routes for further generalization.

In the face of its convenience for theoretical investigations, the definition of data structure used here does not aim at a realistic description of any specific data set. It must be interpreted as a phenomenological or effective parameterization of basic features of data structure that have a distinct effect on linear separability. The limited numerical experiments on MNIST data reported above are a proof of concept, showing a real data set with unexpectedly high linear separability, and they serve as a notable motivation for the investigation of data structure. The main goal of this article is the theoretical analysis; therefore, I postpone any comparison of theory and data. Moreover, MNIST is a relatively simple and clean data set. The numerical analysis signals the highly constrained nature of these data, where points that are close with respect to the Euclidean distance in $R^{n}$ are more likely to have the same label. However, more complex data sets, such as ImageNET, are expected to be less constrained at the level of raw data, due to the higher variability within each category, and due to what are referred to as “nuisances”, i.e., elements that are present, but do not contribute to the classification. Yet, even in these cases, the aggregation of equally-labelled points emerges in the feature spaces towards the last layers of deep neural networks, which improves the efficacy of the linear readout downstream, as empirically observed [14,15].

An interesting, and perhaps unexpected, outcome of the theory concerns the universal properties of the probability of separation $c_{n,p}$. Here, I use the term “universality” in a much weaker sense than what is usually intended in statistical mechanics: I use it to denote (i) the qualitative robustness of the sigmoidal shape of the separability curve on the details of the model, and (ii) the quantitative insensitivity of the separability metrics on all but a few special combinations of parameters [46]. Importantly, the two metrics of data structure that are computed for the model, $p_{c}$ and Δp, are the only two important parameters that fix $c_{n,p}$ in the thermodynamic limit, apart from the rescaling by *k*. The central limit theorem suggests this universality property. In fact, $\gamma_{n,q}$ is the probability distribution of the sum of p−1 independent and identically distributed variables, as expressed by Equation (39). Therefore, $\gamma_{n,q}$ will converge to a Gaussian distribution with linearly increasing mean and variance. This indicates that $\mu_{q}$ and $\sigma_{q}$ are the only two nonzero cumulants in the thermodynamic limit and, thus, $q_{c}$ and Δq are the only two nontrivial metrics that are related to ${\hat{c}}_{n,q}$. This does not, by any means, imply that the model of data structure itself can be reduced to only two degrees of freedom. In fact, the phenomenology is richer if one considers the combinatorial quantity $C_{n,q}$ instead of the intensive one ${\hat{c}}_{n,q}$, see [32]; still, regarding the probability of separation, the relevant metrics are the location and width of the transition region.

## Data Availability

Publicly available datasets were analyzed in this study. The MNIST data set can be found here: yann.lecun.com/exdb/mnist/ (accessed on 20 May 2019).

## Appendix A. Boundary Conditions

The boundary conditions of the recurrence Equation (27) require some care. When a single (q=1) multiplet is considered in dimension n≥k, both its admissible dichotomies are linearly realizable. This is because all dichotomies of *k* points can be realized in n≥k dimensions, as I mentioned above. Therefore

(A1) $${\hat{c}}_{n\geq k,1}=1.$$

The boundary conditions for n<k are not simply the same as for k=1. To see this, consider for instance what happens in n=1 dimensions when dealing with a single (q=1) multiplet of k=2 points, ξ and $\bar{\xi }$. Two problems arise: (i) if the two points lie on opposite sides of the origin, a linearly realized dichotomy ϕ will always assign them different signs, $\phi \left(\xi \right)=-\phi \left(\bar{\xi }\right)$; (ii) there are not enough degrees of freedom to fix the overlap $\rho =\xi \cdot \bar{\xi }$ while keeping ξ and $\bar{\xi }$ normalized.

These obstructions are problematic when trying to define the value of ${\hat{c}}_{1,1}$ for k=2. This quantity appears in the right hand side of the recurrence Equation (25) when n=2 and q=1, where it is needed, alongside ${\hat{c}}_{2,1}$, to compute ${\hat{c}}_{2,2}$. Retracing the derivation for k=2 shows that ${\hat{c}}_{1,1}$ in this context occurs when imposing a linear constraint in 2 dimensions, where it represents the fraction of admissible dichotomies of the doublet $\left\{\xi ,\bar{\xi }\right\}$ that can be realized by a weight versor *w* satisfying $w\cdot \xi^{\prime }=0$. In 2 dimensions, the orthogonality condition fixes *w* up to its sign. If this constrained *w* is such that

(A2) $$\mathrm{sgn}\left(w\cdot \xi \right)=\mathrm{sgn}\left(w\cdot \bar{\xi }\right)$$

then exactly 2 admissible dichotomies of $\left\{\xi ,\bar{\xi }\right\}$ are realizable, otherwise the only realizable dichotomies are not admissible. Therefore ${\hat{c}}_{1,1}$ expresses the probability that Equation (A2) is satisfied; in the mean field approximation, this is $\psi_{2}\left(\rho \right)$. The foregoing argument actually applies for all k≥1. The probability that all *k* points in a multiplet lie in the same half-space with respect to the hyperplane realized by a random versor fixes the first non-trivial boundary condition ${\hat{c}}_{1,1}$.

For k=2 this fixes everything. Let us now consider k=3. In this case Equation (A1) omits ${\hat{c}}_{2,1}$. What should its value be? Again, going back to the argument in Section 6.3 is helpful. ${\hat{c}}_{2,1}$ appears in the recurrence when n=3 and a linear constraint is imposed on *w*. This fixes *w* up to rotations around an axis, identified by a versor *v*. Now, whether the multiplet $\left\{\xi^{1},\xi^{2},\xi^{3}\right\}$ allows 2 or 0 admissible dichotomies depends on whether there exists a vector *w* satisfying the constraint and such that $\mathrm{sgn}\left(w\cdot \xi^{1}\right)=\mathrm{sgn}\left(w\cdot \xi^{2}\right)=\mathrm{sgn}\left(w\cdot \xi^{3}\right)$. This happens if and only if the axis of rotation *v* lies outside the solid angle subtended by the three vectors $\xi^{1},\xi^{2},\xi^{3}$. This characterization allows to compute ${\hat{c}}_{2,1}$ by elementary methods of solid geometry. One finds

(A3) $${\hat{c}}_{2,1}=\frac{1}{2\pi }\left(1-\omega_{123}-\omega_{231}-\omega_{312}-\pi \right)\;\left[k=3\right],$$

where

(A4) $$\omega_{abc}=\arccos\left(\frac{\rho_{a}-\rho_{b}\rho_{c}}{\sqrt{\left(1-\rho_{b}^{2}\right)\left(1-\rho_{c}^{2}\right)}}\right).$$

For larger values of *k*, the same reasoning allows to express the non trivial boundary conditions ${\hat{c}}_{n<k,1}$ as geometric probabilities. Fortunately, the hassle of computing all these probabilities can be bypassed by using the boundary conditions (20), which are approximate for k>1, but still provide asymptotically correct results [28]. In fact, as is evident from the discussion in Section 7, if one takes the thermodynamic limit (30) the contribution of the k−1 approximate values of ${\hat{c}}_{n,1}$ becomes negligible. Other ways of taking the thermodynamic limit (e.g., if *k* is extensive in *n*) may not enjoy this simplification, and may require a different analysis of the boundary conditions.
